# Chapter 7: Pharmacogenomics

**DOI:** 10.1371/journal.pcbi.1002817

**Published:** 2012-12-27

**Authors:** Konrad J. Karczewski, Roxana Daneshjou, Russ B. Altman

**Affiliations:** 1 Program in Biomedical Informatics, Stanford University, Stanford, California, United States of America; 2 Department of Genetics, Stanford University, Stanford, California, United States of America; 3 Department of Medicine, Stanford University, Stanford, California, United States of America; Whitehead Institute, United States of America; University of Maryland, Baltimore County, United States of America

## Abstract

There is great variation in drug-response phenotypes, and a “one size fits all” paradigm for drug delivery is flawed. Pharmacogenomics is the study of how human genetic information impacts drug response, and it aims to improve efficacy and reduced side effects. In this article, we provide an overview of pharmacogenetics, including pharmacokinetics (PK), pharmacodynamics (PD), gene and pathway interactions, and off-target effects. We describe methods for discovering genetic factors in drug response, including genome-wide association studies (GWAS), expression analysis, and other methods such as chemoinformatics and natural language processing (NLP). We cover the practical applications of pharmacogenomics both in the pharmaceutical industry and in a clinical setting. In drug discovery, pharmacogenomics can be used to aid lead identification, anticipate adverse events, and assist in drug repurposing efforts. Moreover, pharmacogenomic discoveries show promise as important elements of physician decision support. Finally, we consider the ethical, regulatory, and reimbursement challenges that remain for the clinical implementation of pharmacogenomics.

**What to Learn in This Chapter**

- Interactions between drugs (small molecules) and genes (proteins)
- Methods for pharmacogenomic discovery
  - Association- and expression-based methods
  - Cheminformatics and pathway-based methods
- Database resources for pharmacogenomic discovery and application (PharmGKB)
- Applications of pharmacogenomics into a clinical setting

This article is part of the “Translational Bioinformatics” collection for *PLOS Computational Biology*.

## 1. Introduction

A child with leukemia goes to the doctor's office to be treated. The oncologist has decided to use mercaptopurine, a drug with a narrow therapeutic range. The efficacy and toxicity of this drug lies in its ability to act as a myelosuppressant, which means it suppresses white and red blood cell production. Despite the dangers this regimen poses, the oncologist is confident with his ability to administer the drug based on his experience with prior patients. However, after the child has undergone treatment, he begins experiencing unexpected bone marrow toxicity, immunosuppression, and life-threatening infections. This type of scenario was encountered after mercaptopurine first came on the market in the 1950s. In the mid-1990s, scientists began to realize that genetics could explain a majority of the cases of life-threatening bone marrow toxicity [1]. Now, many drugs that were once noted to cause so-called “unpredictable” reactions are being re-evaluated for drug-gene interactions.

The history of medicine is full of medications with unintended consequences; the ability to understand some of the underlying causes has been a recent development. In the 1950s, succinylcholine was used by anesthesiologists as a muscle relaxant during operations. However, about 1 in 2500 individuals experienced a horrific reaction – respiratory arrest. Later research revealed that those individuals had defects in both copies of cholinesterase, the enzyme required to metabolize succinylcholine into an inactive form. During the 1980s, a drug used to treat angina, perhexiline, caused neural and liver toxicity in a subset of patients. Scientists later found that this toxicity occurred in individuals with a rare polymorphism of CYP2D6, an enzyme involved in the drug's metabolism. Genetics not only plays a role in adverse events, but also influences an individual's optimal drug dose. Two anticoagulants, warfarin and clopidogrel, have different therapeutic doses based on an individual's genetic makeup. Scientists are increasingly learning more about the interaction between drugs and human genetics in order to take modern medicine down a more personalized path.

Modern physicians prescribe medications based on clinical judgment or evidence from clinical trials. In order to select a drug and dosage, physicians take clinical factors such as gender, weight, or organ function into consideration. The personal variation that may affect drug selection or dosing, such as genetics, is not considered in many settings. Thus, while a daily 75 mg dose of clopidogrel for a 70 kg adult would obviously be inappropriate for a 20 kg child, it is less obvious that two adults with identical presentations and clinical backgrounds might require vastly different doses. However, for an increasing number of drugs, this appears to be the case. For instance, two patients with similar clinical presentations could be given the same dose of the anti-platelet drug clopidogrel, and one would be adequately protected against cardiovascular events while the other experiences a myocardial infarction due to inadequate therapeutic protection. What accounts for this difference? Genetics – the patient with the inadequate therapeutic protection likely has a polymorphism of CYP2C19 with decreased activity, so that this key enzyme cannot efficiently metabolize clopidogrel into its active metabolite. The interaction between drugs and genetics has been termed pharmacogenomics.

In general, pharmacogenomics can be defined as the sum of the word's parts: the study and application of genetic factors (often in a high-throughput, genomic fashion) relating to the body's response to drugs, or pharmacology (for the major questions in the field of pharmacogenomics, see Box 1). Once a patient takes a drug, the drug must travel through the body to its target(s), act on its target(s), and then leave the body. The first and last of these processes is facilitated by pharmacokinetic (PK) genes, which may affect a drug in the “ADME” processes: to be **a**bsorbed into and **d**istributed through the body, **m**etabolized (either to an active form or broken down into an inactive form), and **e**xcreted. The action of a drug on its targets involves pharmacodynamic (PD) genes, which include the direct targets themselves, genes affected downstream, and the genes responsible for the clinical outcome. PK and PD genes can be involved in both intentional “on-target” effects that produce the desired therapeutic response, as well as unintentional “off-target” effects that cause adverse events (side effects or other unintended consequences of the drug). Current researchers are working to tease out genes involved in both the PK and PD pathways that affect drug action in order to improve dosing and avoid adverse drug reactions.

**Box 1. Problem Statement**

- What are the genes involved in a drug's mechanism of action?
- How are a drug's effects propagated through pathways?
- How can this information be applied to characterize “off-target” adverse events?
- How can pharmacogenomics information be utilized in prescription and dosing decisions?

The search for genetic factors that relate to pharmacological response begins much like the search for a genetic association of any trait. Standard association study methods (such as GWAS) search for significant associations between a binary or continuous trait and the genetic profiles of case and control sets. In a GWAS, the trait of interest can be a disease state or physical trait. Specifically, in the case of pharmacogenomics, the trait is an actual drug dose, response, or adverse event profile, though the study design should be carefully considered for the specific application (see below: Methods). Additionally, high-throughput expression analysis and cheminformatics have provided investigators with valuable tools for learning about physiological drug responses. Finally, as sequencing technologies become exponentially cheaper and the “$1,000 Genome” becomes an attainable goal, whole-genome or exome sequencing will soon become commonplace in pharmacogenomic studies. As these types of studies become less expensive and more mainstream, pharmacogenomics will transition from simply an interesting research topic to a main role player in pharmacological development and clinical application.

The applications of pharmacogenomics are of interest to industry, clinicians, academics, and patients alike. For the biopharmaceutical industry, pharmacogenomics can improve the drug development process through faster and safer drug trials and the early identification of drug responders, non-responders, and those prone to adverse events. For clinicians and patients, pharmacogenomics can aid the decision-making process in prescriptions and determination of the optimal dose of a drug.

Many significant challenges remain in the field of pharmacogenomics, beyond the simple identification of more genetic variants related to drug response. First, the transition to whole-genome sequencing will require newer analysis methods, as well as more extensive annotations, to assign meaning to novel variants. A database of the relation between genes, variants, and drugs, such as PharmGKB, will be instrumental in the aggregation of information curated from the literature. In addition, the characterization of adverse events and their underlying causes is a topic of active research. Finally, the application of pharmacogenomics to a clinical setting will require the education of physicians in the utility of genome sequencing or genotyping for the benefit of their patients.

With the dawn of human genome sequencing, especially the impending widespread availability of personal genotyping to the public, and an expanded knowledge of the clinical impact of genetics and molecular biology, physicians around the world are beginning to use patients' personal genetics in informing prescription decisions. While still in its early phases, pharmacogenomics will undoubtedly lead the way in the development of personalized medicine.

## 2. Pharmacogenomics in Action

When a physician administers a drug, an intricate cascade of events unfolds as this molecule interacts with the physiological environment. In the simplest scenario, a drug (after interacting with a number of proteins on its way to its target) may act as an agonist or an antagonist against a receptor, which is composed of one or more proteins. At the molecular level, the metabolite can bind to the protein's active site, which can include ligand-binding sites, conformation-altering sites, or catalytic sites. This effect can then be propagated through biochemical pathways to produce a cellular and finally, systemic physiological effect. Along the way, human genetic variation can affect the way these receptors interact with drugs, leading to consequences in the efficacy of the drug and causing potential adverse events.

### 2.1. Drug-Receptor Interactions: Agonists and Antagonists

Agonists interact with a receptor in an activating fashion: these small molecules mimic the behavior of the receptor's natural ligand, producing a result that is either weaker than, the same as, or stronger than the natural ligand. For example, sympathomimetic drugs are a clinically important class of agonists that interact with the G-protein-coupled receptors that are endogenously stimulated by catecholamines. These drugs are given to produce responses normally elicited by the sympathetic nervous system. Some examples of sympathomimetic drug action include relaxation of bronchial smooth muscle in asthma, increasing the muscular contractions of the heart in cases of reversible heart failure due to cardiogenic or septic shock, or vasoconstriction of superficial vasculature to reduce nasal congestion. There are several subtypes of adrenoreceptors and different drugs stimulate different receptor subtypes. For instance, a very clinically relevant drug, albuterol, can be inhaled to stimulate β2 receptors (whose natural ligand is norepinephrine) on the smooth muscle of the lungs. Its action leads to the activation of adenylyl cyclase, which ultimately leads to the dilation of bronchial smooth muscle, providing life-saving relief for asthma patients (See Chapter 9 of [2]). However, some studies have identified that the very agonists that provide relief to asthmatics can lead to asthma exacerbation or death in a subset of patients. Research has indicated that at least in some populations, this phenomenon could be related to genetic polymorphisms of the β2 receptors [3].

Antagonists, on the other hand, inhibit the receptor partially or fully, reversibly or irreversibly so that the cascade caused by normal receptor activation cannot occur. The same adrenergic receptor subclasses mentioned before can also be antagonized. β receptor blockers (“beta-blockers”) are an antagonist drug class clinically indicated to treat chronic, irreversible heart failure. The mechanism of the beneficial effects of β blockers is not well understood. The prevailing theory is that since the high levels of circulating catecholamines triggered by heart failure lead to detrimental cardiac remodeling, blocking the cardiac catecholamine receptors (β1 and β2 receptors) with a β blocker can slow down additional de-compensation. The β blockers for heart failure, bisoprolol, carvedilol, and metoprolol, antagonize (that is, inhibit) β1 and β2 receptors: their action is substantially greater at the β1 receptor, which is the dominant receptor in the heart. However, some patients do not respond as well to this therapy as others, and clinical studies have suggested that this may be due to β1 receptor polymorphisms. More extensive studies of these polymorphisms are underway to definitively identify the pharmacogenetic variables affecting β blocker success [4].

Often in the literature, the discussion of drugs and proteins has involved vague notions of “interactions” without any discussion about the underlying molecular mechanisms. A drug's interaction with any receptor is dependent on how well the molecular conformation of the drug can interact with the structure of the target. Before any discussion of downstream physiological effects, a drug's mechanism of action begins with the specific molecular reaction between the drug and cellular proteins. This interaction itself can provide insight into the effect of drugs on physiology and influence potential pharmacogenomic knowledge.

### 2.2. Drug-Receptor Interactions: The Details

While biologists tend to represent proteins as colored ovals existing in an idealized environment, in reality, proteins are complex molecules with intricate secondary and tertiary structures: they harbor rugged landscapes on their surfaces, with charged or hydrophobic hills and valleys serving as pockets to which potential small molecules can bind. At these twists and turns, proteins contain their active sites, including structural sites, binding sites, and catalytic sites. Metabolites (drugs) that enter a protein's binding site or catalytic site can either switch on the function of the protein (agonists) or prevent further reactions (antagonists). Such an effect is especially common if the drug bears chemical similarity to the natural ligand of the protein.

Non-steroidal anti-inflammatory drugs (NSAIDs), which cause both reversible and irreversible inhibitory processes, are a familiar drug class that illustrates drug-protein interactions. In general, NSAIDs inhibit the action of cyclooxygenases (coded by the COX genes), which mediate inflammation (see below: Molecular and Physiological Effects; reviewed in [5]). For instance, ibuprofen inhibits cyclooxygenases in a reversible fashion, by localizing to its critical catalytic site and competing with arachidonic acid to prevent the modification of the substrate [6].

Alternatively, a drug can react covalently with a protein's critical structural, binding, or catalytic site to affect the structure of the site or the protein as a whole. As mentioned previously, drugs can covalently modify their protein targets, causing protein inactivation. In the case of NSAIDs, aspirin irreversibly inactivates cyclooxygenases by acetylating critical serine resides (e.g. Serine 530 of COX-2): the bulky sidechain renders the catalytic sites unable to modify arachidonic acid [7]. Irreversible reactions can also work in the opposite direction, where the protein modifies the structure of the drug, potentially altering its activity (see below: PK Interactions).

**Figure 1:**
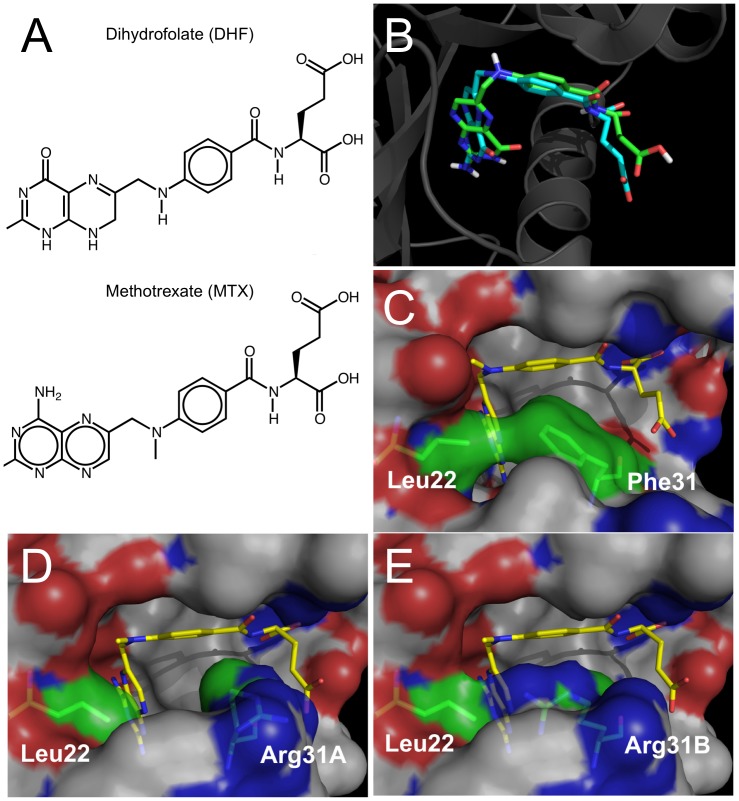
Methotrexate binds to the folate-binding region of DHFR. (A) Structural similarity between methotrexate and dihydrofolate. (B) Methotrexate (green) and dihydrofolate (blue) fit into the same binding pocket of DHFR. (C) The conformation of dihydrofolate bound to the reference version of the receptor. (D–E) Two possible conformations of dihydrofolate bound to the F31R/Q35E variants of the receptor. These variants have decreased affinity to methotrexate, relative to dihydrofolate. Reprinted with permission from [8].

Often, such an interaction occurs because a drug bears structural similarity to the molecule's natural ligand. For instance, methotrexate is an antifolate drug used to treat a number of diseases, including cancers and autoimmune diseases. Methotrexate is structurally similar to dihydrofolate (Figure 1A) and as such, binds to the same region of DHFR (Figure 1B). Dihydrofolate typically fits into DHFR in a known conformation (Figure 1C), but a phenylalanine to arginine mutation changes this binding conformation (Figure 1D–E). This mutation is hypothesized to confer methotrexate resistance in individuals with this variant [8].

All such drug-protein interactions are often associated with the “intended” action of the drug, whether they involve “what the body does to the drug” (pharmacokinetics, PK) or “what the drug does to the body” (pharmacodynamics, PD). However, drug-protein interactions may also lead to “off-target” interactions, which can cause adverse events. Along the way, variants in genes can affect these interactions, which influence the pharmacological effect of the drug (See Figure 2 of [9]).

**Figure 2:**
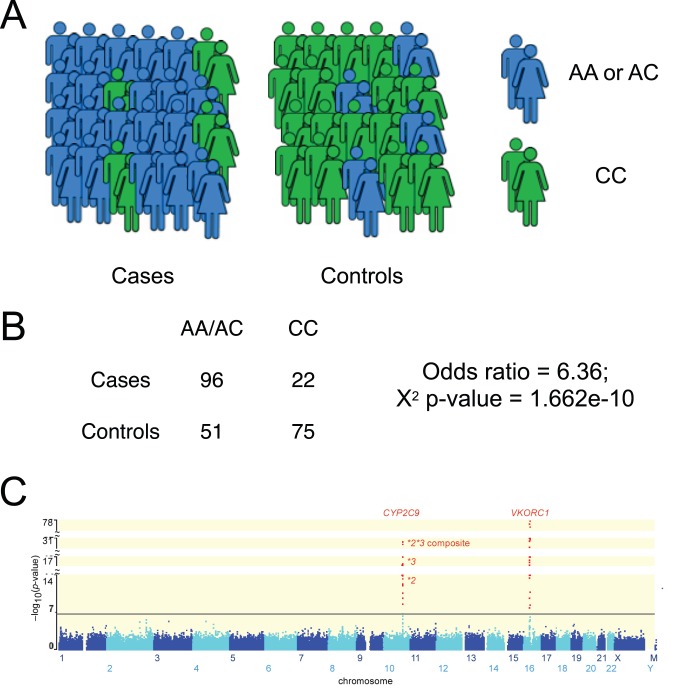
Association methods. (A) An association study with cases and controls. Millions of genetic loci are probed to ascertain “association,” or separation between genotypes in cases and controls. (B) Each SNP is tested independently using a 2×2 contingency table and a χ^2^ test or Fisher's exact test. (C) Each SNP is assessed for “genome-wide” significance, after Bonferroni correction. Reprinted from [64].

#### 2.2.1. Pharmacokinetic (PK) interactions

On the way to its target and on its way out, a drug may interact with many proteins that aid or hinder its progress. These interactions define a drug's pharmacokinetics, which encompass absorption, distribution, metabolism, and excretion (ADME) processes. These parameters determine how quickly a drug reaches its target and how long its action can last.

When a drug is administered, it must first be absorbed by the body and distributed to the relevant organs and cells. One important parameter, bioavailability, involves the fraction of the dose of the drug that ends in systemic circulation, much of which is based on mode of administration: intravenous delivery would provide 100% bioavailability, while an orally ingested tablet or capsule may be incompletely absorbed by the gastrointestinal tract or metabolized before it reaches systemic circulation. For non-injection methods (as most prescription drugs are administered), bioavailability often depends on absorption and enzymatic action. If the drug is administered orally, bioavailability is influenced by gastric emptying (i.e. transit time), gastrointestinal enzymatic action, gastrointestinal absorption, and liver metabolism. Since drugs absorbed from the gastrointestinal system are taken to the liver via the portal vein prior to entering systemic circulation, the liver can exert a tremendous effect on first pass metabolism. Once a drug has entered systemic circulation, issues of molecular transport affects the drug's ability to distribute (or reach its target). Genetic variation in the proteins that mediate these processes can affect the absorption and distribution of certain drugs. For instance, the class of ABC (ATP binding cassette) transporters is involved in many of the transport processes in the circulation of drugs and metabolites, especially in the gut and across the blood-brain barrier: polymorphisms in these genes is associated with altered bioavailability of certain drugs, such as the cardiac drug digoxin (digitalis; reviewed in [10]).

The body's metabolism of a drug can lead to the conversion of a precursor drug into an active metabolite or the breakdown of the active form into an inactive form for excretion. As with absorption and distribution, inter-individual variation in metabolism can often be explained by genetics (specifically, changes in the proteins that interact with the drug). Perhaps the most famous drug-metabolizing proteins are members of the cytochrome P450 family (“CYP” genes), which are involved in the phase I metabolism of the majority of known drugs [11]. Polymorphisms in these genes have been implicated in human drug response variation, affecting up to 25% of all drug therapies (reviewed in [12]). For instance, CYP2C9 plays a major role in the metabolism of warfarin to the inactive hydroxylated forms, including 7-hydroxywarfarin ([13], reviewed in [14]). As such, CYP2C9 is the second greatest contributor to the variation in warfarin dosage discovered thus far, which has led to its inclusion in pharmacogenetic dosing equations [15].

Finally, the body constantly cycles through the gamut of small molecules that flow through it. For example, the kidney is involved in finely regulating ionic concentrations and purging out unwanted metabolites. As small molecules, drugs are not exempt from these processes and are also excreted from the body, purging what was brought in and circulated by absorption and distribution. For instance, one member of the ABC family, P-glycoprotein (P-gp or ABCB1) is a transporter protein that actively pumps drugs and other metabolites out of cells (a detailed view into the mechanism of P-gp can be found in [16]). Upregulation of P-gp causes increased efflux of small molecules, which causes multi-drug resistance. For example, resistance to statins and chemotherapeutic drugs occurs because the drugs are pumped out before achieving their therapeutic effect (reviewed in [17]). Thus, inhibition of P-gp has remained an active area of research for augmenting cancer treatment [18]. Additionally, upregulation of elimination mediators such as P-gp should be considered for pharmacogenomic dose adjustments, with the caveat that increasing a drug's dose may have other potential detrimental effects.

#### 2.2.2. Pharmacodynamic (PD) interactions

Pharmacodynamics (PD) encapsulates the specific effect of the drug on its targets and downstream pathways. The drug-target interactions can be “on-target”, where interactions lead to a therapeutic effect, or “off-target”, where interactions lead to undesired effects. PD also deals with how a drug concentration affects the target – what concentration is needed to reach the maximum effect, beyond which additional drug does not increase response (maximal effect) and what concentration is required to reach half of this maximal effect (sensitivity).

In many cases, structurally similar molecules (e.g. a drug that is similar to a protein's natural ligand) can bind and affect the same region of a protein and produce a pharmacological effect. For instance, vitamin K and warfarin both interact with VKORC1 (Vitamin K epOxide Reductase Complex subunit 1), an enzyme that typically converts the inactive epoxidized form of Vitamin K back to the active reduced form [19]. Warfarin binds to VKORC1 near its catalytic site (See Figure 3 of [20]), inhibiting the reduction reaction; the ensuing lack of active Vitamin K results in the downstream anti-coagulant effects of warfarin (See Figure 2 of [21] and below: Molecular and Physiological Effects). Polymorphisms in VKORC1 are intensely linked to the efficacy of warfarin [22] by affecting warfarin's ability to bind to VKORC1 and displace vitamin K. As such, sensitivity to warfarin varies significantly in individuals, leading to twenty-fold dose differences. Warfarin's optimal dose can be better estimated by including VKORC1 polymorphisms in a dosing equation rather than using clinical factors alone [15].

**Figure 3:**
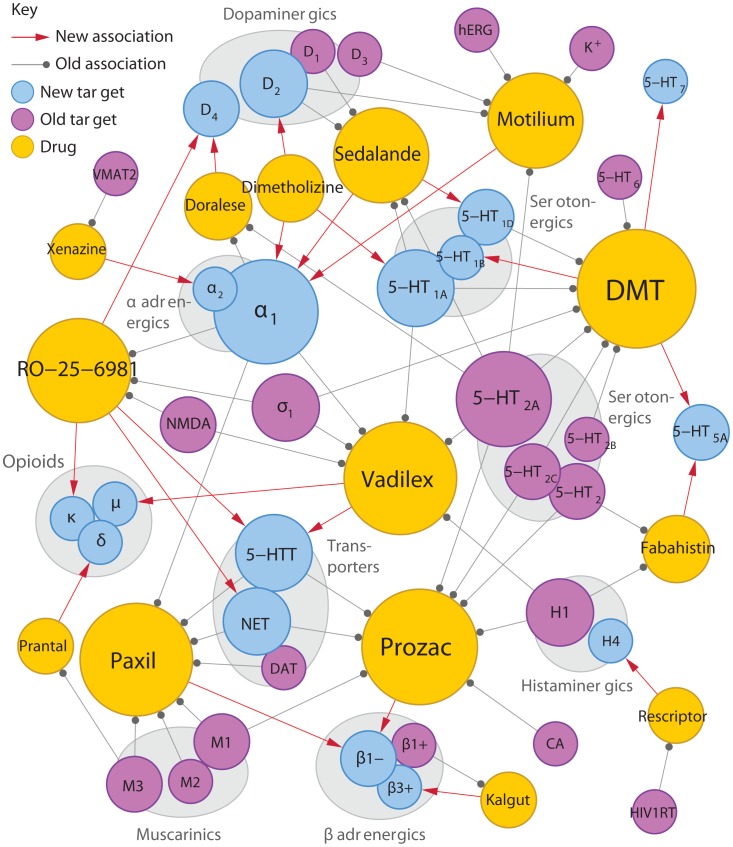
Cheminformatics methods. New associations discovered by cheminformatics methods. The Similarity Ensemble Approach (SEA) uses ligand similarity methods to discover potential new associations between drugs and targets. Reprinted with permission from [33].

Often, a drug's mechanism of action involves its localization to some binding pocket that then disrupts (or enhances) the function of the protein. For example, hydrocortisone is a lipid-soluble drug that diffuses across the cell membrane and interacts with the glucocorticoid receptors. These receptors reside in an inactive conformation because they are bound to heat shock proteins, which hold the glucocorticoid receptors in the inactive state. The binding of hydrocortisone causes the dissociation of the heat shock protein and allows the DNA-binding and transcription-activating binding domains of the glucocorticoid receptor to enter an active conformation. Now, target genes can be transcribed, and the many anti-inflammatory downstream effects of hydrocortisone can occur (See Chapter 2 of [2]).

### 2.3. Propagation through Pathways

As in the example of hydrocortisone, once a drug affects a gene (whether “on-target” or “off-target”), the effects can propagate through multiple proteins in the same pathway. Biology does not occur in a vacuum: proteins are dynamic and interact with many other proteins to produce a physiological function.

In the simplest cases, if the direct effect of a drug is the inhibition of a functional protein, all downstream effects of that protein will be affected. For instance, if a drug disrupts a kinase's active site, all downstream factors in a kinase cascade would not be phosphorylated. As in the case of hydrocortisone, a drug's activation of a transcription factor's DNA-binding domain will switch on the expression of the transcription factor's targets. These downstream targets lead to many of the biological effects of a given drug. Thus, a variant in a pharmacogene may be considerably upstream or downstream of the drug's direct protein interactions, but still affect the action of the drug.

For instance, suppose protein A is known to interact with proteins B and C. When a drug is used to block protein A in order to inhibit protein B's downstream effects, the interaction between proteins A and C may also be affected. If protein A and C's interaction is essential for healthy cellular function, administration of the drug could lead to severe adverse events. Most of the interactions discussed so far comprise “on-target” effects (A and B), while “innocent bystander” interactions (A and C) are known as “off-target” events. In other cases, the drug may exert an effect on an unrelated protein D (that may, for example, bear structural resemblance to protein A).

### 2.4. Adverse Events (“Off-Target”)

Drugs are designed for their therapeutic effects, which require the molecule to bind to one or more targets that then produce downstream effects. Adverse events, however, can occur when the “on-target” interaction produces a potentially related, but unintended effect, or when drugs bind to “off-target” proteins to produce an unrelated, unintended effect. Such effects may be harmful to the patient, but may occasionally be inadvertently helpful (see below: Drug Repurposing). For instance, this adverse event can occur due to the intended interaction in an unintended tissue: the β blockers used to treat heart failure can also block β receptors in the bronchial smooth muscle, causing bronchial spasm, a dangerous event for asthmatics (See Chapter 13 of [2]). Another example is tamoxifen, the selective estrogen receptor modulator (SERM), which has improved outcomes in patients with estrogen receptor positive breast cancers. This drug antagonizes the estrogen receptor in the breast, blocking one of the signals that the cancer cells rely on. However, tamoxifen also has agonist activity at the estrogen receptors in endometrial tissue. This off target action can lead to a 2- to 7-fold increased risk of endometrial cancer [23].

Alternatively, a drug may interact with a protein (unrelated to the intended target) to produce an “off-target” adverse event. For example, in addition to the “on-target” adverse events described above, tamoxifen is also associated with cardiac abnormalities and muscle cramping. Preliminary data (discovered by docking methods, see below: Cheminformatics) suggest that these events may be due to an “off-target” interaction with sarcoplasmic reticulum Ca^2+^ ion channel ATPase protein (SERCA) [24].

### 2.5. Molecular and Physiological Effects

A drug's interaction with its target and the downstream effects (through any of the target's pathways) leads to the alterations in cellular physiology. In some cases, a cellular “systemic” response may be activated or switched off, such as apoptosis or inflammation. The cell may signal to other cells to produce a larger response, which is then observed in the larger context of the body. For instance, warfarin's inhibition of VKORC1 slows the vitamin K-dependent clotting pathway. This results in decreased thrombus formation by platelets, or colloquially known as “blood thinning.” In other cases, a drug may suppress a body's natural response. For instance, NSAIDs such as aspirin and ibuprofen inhibit COX proteins, preventing the conversion of arachidonic acid to prostoglandin H_2_ (PGH_2_) and blocking the downstream production of other prostoglandins, which mediate inflammation and pain response.

While in the case of VKORC1, pharmacogenomic variation is observed at the direct site of action of warfarin, variation in downstream receptors can also influence the effect of drugs on the body. For instance, calumenin (CALU) is an inhibitor of the vitamin K-dependent clotting pathway. While calumenin's effects are downstream of the direct interaction between VKORC1 and warfarin, variants in calumenin are also associated with differences in warfarin dosage [25].

## 3. Methods for Discovery of Pharmacogenomic Genes and Variants

Pharmacogenomic research aims to identify the genes (and gene variants) involved in the interaction between a drug and the body. For any of the pharmacogenomic applications discussed below, there exist methods for discovering relevant genes and variants (typically single nucleotide polymorphisms, or SNPs) related to drug response. Traditional SNP-based methods, such as genome wide association studies (GWAS), can be used to discover candidate regions of interest. Alternatively, analysis of other sources of data, including expression or biochemical data, may provide additional gene candidates. Once candidate variants are identified, further computational and experimental follow-up may be required to fully characterize all the genes and pathways involved in the drug's progression through the body.

### 3.1. Association Methods

In a GWAS, hundreds of thousands or millions of SNPs (representing regions of the genome with the most inter-individual variation) are probed on a DNA microarray for each individual in a set of cases and controls (Figure 2A). For each SNP, significance of the association between a SNP and the trait is measured a chi-squared test, based on a 2×2 contingency table of alleles (or genotypes, if a dominant or recessive model is assumed) and case/control status (Figure 2B). In the case of a continuous independent variable, such as drug dose, a likelihood-ratio test or a Wald test is applied to measure whether there is a significantly different dose between the two groups of genotypes.

Each SNP is tested independently, and thus significance (p-values) must be corrected for multiple hypotheses, usually using a Bonferroni correction or False Discovery Rate (FDR). A SNP that reaches “genome-wide significance” (Figure 2C) is then a candidate for follow-up analysis, as are genes in or near the significant SNP, genes for which the SNP is an eQTL (a SNP associated with the expression of some other gene), and genes in the same pathway as these genes.

The two most important considerations for the design of any pharmacogenomic study include the selection of representative genetic markers, as well as phenotypically well-characterized patients (including cases and controls). The first of these, design of a suitable genotyping array, is technically easy and inexpensive, though the exact design can depend on the desired balance between unbiased genome-wide studies and a targeted SNP panel (see below). As in any trait-association study, the second consideration: the selection, characterization, and covariate identification of cases and controls provides a significant challenge.

Because performing a million independent tests requires stringent significance correction, large numbers of cases and controls, often in the thousands, are required to discover a SNP that will achieve “genome-wide” significance. SNP-based GWAS methods are effective when there is a strong signal from some SNP for the size of the study (that is, when there is good separation between genotypes for the cases and controls). However, under this stringent independence model, weaker signals may be lost among the noise that plagues genetic association studies. Thus, combining data from multiple SNPs in a single gene can boost power and decrease the number of hypotheses for multiple hypothesis correction [26]. Alternatively, if we have prior information about the drug's mechanism of action, we can create targeted SNP panels, limited to genes in the drug target's pathway, to decrease the hypothesis space [27].

As the price of high-throughput sequencing continues to fall, many investigators are turning to exome or whole genome sequencing to discover genetic factors of drug response. Such technologies have the advantage of remaining unbiased in SNP discovery, detecting less common (and even personal) mutations, and capturing larger-scale information, including copy number variants (CNVs) and structural variants (SVs).

Often, in major association studies, the SNP platform (DNA microarray) used is comprised of SNPs that serve as “tags” for a larger stretch of nearby SNPs. Such an approach is possible due to the presence of “linkage disequilibrium” in the genome, a phenomenon where SNPs tend to be inherited together (“linked”); the particular structure of these “haploblocks” (which SNPs are typically inherited together) is specific to each racial population. Because different populations have different linkage structures and a different series of polymorphisms, platforms that are optimized for one population may not be the best choice for another. This problem is further complicated by underlying differences in genomes: the effect a given SNP has on drug response may be different (or even the opposite) because of a hidden interaction with an alternate variant of another gene. Specifically, since many of the original genotyping platforms were developed for Caucasian populations, studies on Africans or Asians will require different approaches. Additionally, the first SNP identified is typically simply an “associated” variant, rather than the causative variant. In order to determine the specific proteins directly involved in drug response, further experimental or informatics analysis must be performed on genes and variants “linked” to the associated variant.

### 3.2. Expression Methods

In addition, other sources of data can be used to identify genes involved in drug response, including RNA expression data from microarrays or RNA-Seq experiments from drug-treated samples. For instance, using expression profiles from patients with a disease of interest, one can identify the genes involved in the progression of the disease and identify potential drug target candidates. Alternatively, expression profiles generated from a drug treated sample (compared to control) can be used to determine a molecular response to a drug. Ideally, such drug treatment experiments would be done on humans in order to generate organic in vivo physiological response. However, such experiments are unethical for experimental (early phase) drugs, require significant regulatory approval, and are expensive. Thus, established cell lines have provided a valuable, lower-cost resource for investigators to generate gene expression profiles.

One such effort, the connectivity map (CMAP), a publicly available resource of gene expression data of cell lines treated with various small molecules, has been used to compare expression profiles (See Figure 1 of [28]) to identify metabolite-protein interactions, small molecules with similar binding profiles, and metabolites that may mimic or suppress disease [28]. For instance, this approach predicted gedunin to be an inhibitor of HSP90 due to the similarity between gedunin's expression profile and the profile of known inhibitors. Despite the lack of structural similarity between gedunin and other HSP90 inhibitors, CMAP's predicted result was validated biochemically.

Thus, cell lines can be used as surrogates for individuals, where a cellular phenotype is used as a proxy for the individual's own physiological response based on the cellular expression response to a drug treatment. For instance, one can search for associations between a cell line's genetic makeup and cell viability after drug treatment (the IC_50_ of drug for each cell lines) [29]. Alternatively, similar methods can be used to characterize toxicological response: treating cell lines with a drug and measuring gene expression can suggest genes involved in the drug's toxicity.

While not yet extensively employed in practice, other sources of high-throughput experimental “omics” data, such as metabolomics or proteomics data could be used for similar analyses.

### 3.3. Cheminformatics/NLP (Other Discovery Methods)

While not strictly “pharmacogenomics” methods, cheminformatics has provided a valuable tool for investigators in the initial stages of drug discovery. For instance, combining information about protein structure and small molecule structure, docking methods predict the best fit of a molecule (or all molecules in a database such as PubChem or ChEMBL) by minimizing the conformation energy of the molecule-protein “fit”. Such methods are computationally expensive, as they explore a large search space for each pair of molecule and protein and use molecular dynamics or genetic algorithms to optimize fits. Therefore, molecule docking can be limited to the active site of a protein with a group of molecules to decrease the search space. Alternatively, if a given molecule is previously known to interact with a protein, molecule similarity metrics can be included to suggest similar molecules as protein-binding candidates. In this way, a search limited to ligands that score above a similarity threshold to the known ligand would be much faster than a search through all of PubChem. While such predictions must still be confirmed through biochemistry (such as binding assays), these methods can be used to limit the hypothesis space for drug discovery, prioritizing the expensive, lower-throughput biochemical assays.

For a potential drug target, cheminformatics methods can be used to identify new “hits” or optimize “leads” by suggesting molecules that may disrupt the function of the protein. For instance, docking methods were used to successfully identify novel molecules that could serve as inhibitors of CTX-M β-lactamase at millimolar binding affinities [30]. Various algorithms have been developed for screening ligand-target fits using docking (reviewed in [31]). Additionally, methods that incorporate the structure of the ligand along with known interactions can identify patterns of related drug targets [32]. Such an approach can suggest new functions for known drugs, explain “off-target” adverse events, and importantly, predict “polypharmacology,” or the action of a single drug on multiple targets [33] (Figure 3). These methods leverage small molecule databases such as PubChem and ChEMBL, which maintain structures and properties of small molecules and ligands, as well as bioassay results of these compounds.

Additionally, a wealth of scientific information is available in the biomedical literature as lower-throughput free text. Thus, text mining techniques such as natural language processing (NLP), which exploits sentence syntax to pull structured knowledge from the literature, can be used to mine PubMed and other sources of published information to discover new drug-protein interactions [34].

### 3.4. Pathway Discovery

Once a candidate gene is identified, studying the gene's known genetic networks, cascades, and pathways can help identify other possible candidates that affect drug action. For instance, if a kinase is identified as a drug target, the proteins it phosphorylates (and any proteins affected downstream) may be relevant to the study of the drug. Additionally, knowledge of biological pathways influencing a disease can aid in the drug discovery process (see below: Drug Discovery).

Numerous online or downloadable resources exist for pathway and network analysis, such as Biocarta, Ingenuity, KEGG, and PharmGKB. For a gene-drug relationship of interest, information on the gene's network or pathway can be used to limit the hypothesis space of other analyses and experiments. Pathway analysis can “connect the dots” between known gene-drug interactions to generate new hypotheses of key genes that may also contribute to the pharmacogenomics of the drug. Additionally, a mechanism of action can be formalized by closing the loop between all the genes involved.

### 3.5. Validation and Application

The methods discussed thus far provide only computational evidence for potential drug-protein interactions. In order to prove drug-protein interactions and effects, follow-up biochemical methods, such as measuring binding affinity or functional assays, are required to demonstrate a molecule's potential therapeutic activity or to definitively prove an interaction.

Ideally, multiple sources of evidence can be integrated to fully characterize the physiological response to a drug. Once sufficient confidence is generated for a potential pharmacogenomic mechanism, the first step towards clinical application involves the storage and dissemination of the information in a curated database, such as PharmGKB. Combining information from multiple analyses will allow for more powerful characterization of the pharmacogenomic response. For instance, dosing equations for sensitive drugs such as warfarin can be developed by multiple linear regression of variants (as well as clinical covariates) on observed doses [15]. Finally, a centralized resource such as PharmGKB will allow for systematic pharmacogenomic analysis: such as for automated annotation of an input of genomic variants.

## 4. Pharmacogenomics in Drug Discovery

**Figure 4:**
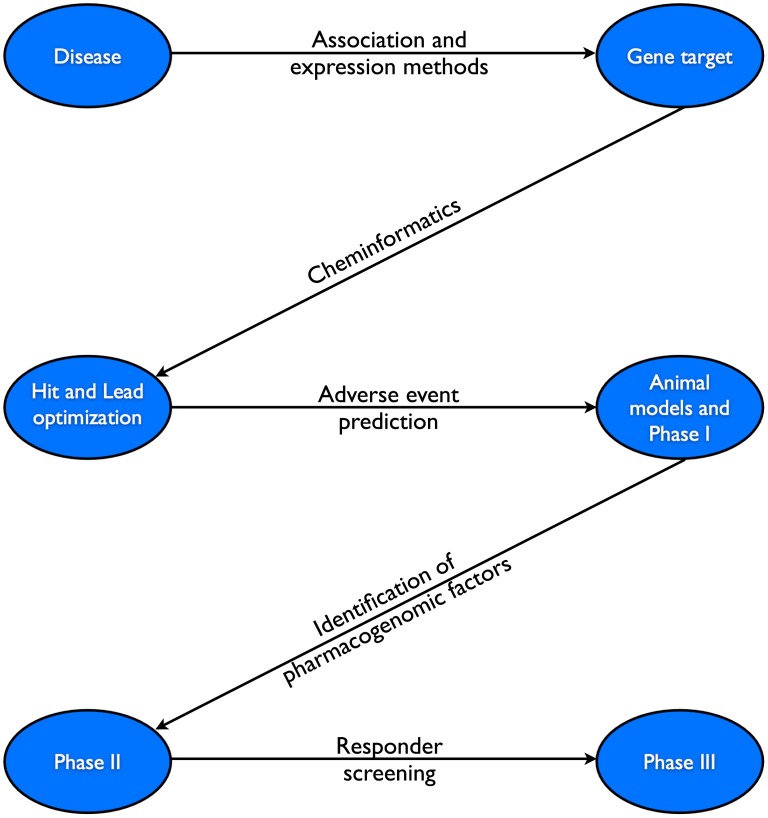
Drug discovery. Pharmacogenomics can be used at multiple steps along the drug discovery pipeline to minimize costs, as well as increase throughput and safety. First, association and expression methods (as well as pathway analysis) can be used to identify potential gene targets for a given disease. Cheminformatics can then be used to narrow the number of targets to be tested biochemically, as well as identifying potential polypharmacological factors that could contribute to adverse events. After initial trials (including animal models and Phase I trials), pharmacogenomics can identify variants that may potentially affect dosing and efficacy. This information can then be used in designing a larger Phase III clinical trial, excluding “non-responding” and targeting the drug towards those more likely to respond favorably.

Pharmacogenomics can impact how the pharmaceutical industry develops drugs, as early as the drug discovery process itself (Figure 4). First, cheminformatics and pathway analysis can aid in the discovery of suitable gene targets, followed by small molecules as “leads” for potential drugs. Additionally, discovery of pharmacogenomic variants for the design of clinical trials can allow for safer, more successful passage of drugs through the pharmaceutical pipeline.

### 4.1. Small Molecule Candidate Identification

A key starting point in developing a drug for illness or disease involves finding a suitable gene to target. Typically, genes implicated in a disease can be discovered by GWAS, exome sequencing, analysis of RNA expression profiles, or other biochemical methods. These genes and others in the same pathway can be considered as candidate drug targets. The potential target space could be limited by excluding genes on the basis of their similarity to other genes (possibly due to paralogy) to avoid “off-target” effects.

Once potential gene or pathway targets are identified, cheminformatics methods can be used to generate predictions for potential “leads” (or drug candidates) for a high-throughput drug screen. For instance, protein structure information can be combined with small molecule structure information to predict favorable drug-gene interactions. After such predictions are generated, follow-up biochemical experiments would be required to confirm the interaction before the small molecules are considered further.

In a similar vein, pathway analysis can be used to select new, potentially safer drug targets. Namely, if a drug (which targets some gene) is initially discovered as effective, but found to cause adverse events, safer alternatives might be found by searching for drugs that target genes in the same pathway as the original gene.

### 4.2. Clinical Trial Pipeline

Once a small molecule has been biochemically identified as a “lead” and a lack of toxicity verified in animal models, the small molecule goes through a series of increasingly larger phases of clinical trials. Basic efficacy and relative safety are demonstrated before and during Phase II clinical trials, on the path to Phase III. However, Phase III trials often require thousands of patients, and thus, a pharmaceutical company would ideally be confident that the drug will successfully pass and be profitable before investing in such an expensive endeavor.

Most of the time, patient response to a drug is variable during the initial Phase II trials and as this response is often related to genetic factors (PK or PD protein variability), pharmacogenomics can be used to limit the cohort for Phase III trials. Specifically, if a protein variant is identified that separates drug responders from non-responders, individuals with the “non-responder” variant could be excluded from the next phase of the trial (reviewed in [35]). While this would limit the scope and usability of the drug, it would ensure the passage of the drug through the trial. As such, pharmaceutical companies would need to balance the loss in revenue of a less globally applicable drug with the risk of FDA rejection of the drug.

### 4.3. Drug Repurposing

As mentioned previously, cheminformatics methods can be used to identify novel drug-protein interactions. While these predicted interactions can be used to discover new small molecules for therapeutic purposes, any new drug must still go through the significant regulatory hurdles of safety and efficacy testing. However, drugs already on the market for some therapeutic purpose are FDA-approved for safe use in humans, and their “repurposing” would simply involve demonstrating that the drug can be used effectively for a different indication. In general, any method that can be used to characterize “off-target” effects can be used in drug repurposing, by finding effects that are salubrious.

**Figure 5:**
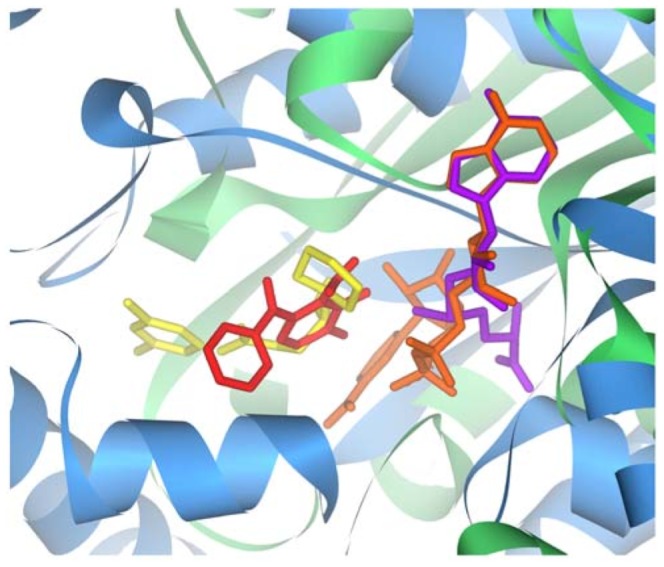
Drug repurposing. Docking methods suggest binding site similarity between COMT (green) and InhA (blue). The overlap between the predicted locations of their cofactors (purple and orange, respectively) and ligands (red and yellow, respectively) suggest potential similarity in their functions. Thus, the same drug that has been used to inhibit COMT (entacapone) was predicted to inhibit the M. tuberculosis protein InhA for potential treatment of tuberculosis. Reprinted from [36].

For instance, docking methods have been used in discovering novel functions for already-established small molecules (or drug “repurposing” or “repositioning”). The similarity between a drug target for Parkinson's disease, catchol-O-methyltransferase (COMT) and a bacterial protein in Mycobaterium tuberculosis (the enoyl-acyl carrier protein reductase, InhA) narrowed down an investigation of potential drug targets for M. tuberculosis infections (Figure 5). From this result, entacapone, a drug already approved to treat Parkinson's by inhibiting COMT, was predicted to bind to InhA, which was then validated biochemically and shown to have antibacterial activity [36]. Thus, while full efficacy for treatment of tuberculosis must still be demonstrated in larger studies, studies on a known safe drug are significantly cheaper and carry much lower risk.

## 5. Applying Pharmacogenomic Knowledge

Pharmacogenomics has the potential to transform the way medicine is practiced, by replacing broad methods of screening and treatment with a more personalized approach that takes into account both clinical factors and the patient's genetics. As demonstrated previously, genetic variation can greatly influence the nature of the effects a drug will have on an individual (whether it will work or cause an adverse event), as well as the amount of drug required to produce the desired effect. To this end, pharmacogenomics will impact the way drugs are prescribed, dosed, and monitored for adverse reactions.

**Figure 6:**
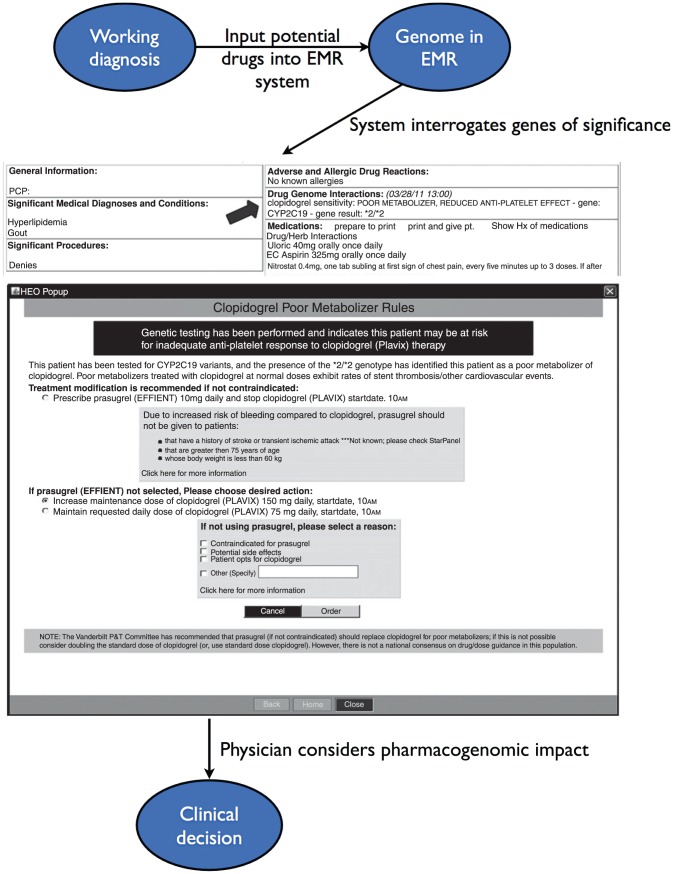
Applying pharmacogenomics in the clinic. A proposed clinical workflow including pharmacogenomic information. A physician considers the patient's current presentation and past history when coming up with a working diagnosis and based on his or her clinical judgment, decides what drugs the patient may need. For example, if the physician wanted to add clopidogrel to the patient's regimen, the physician would input it into the electronic medical record (EMR). The EMR would interrogate the genome and present a message such as “clopidogrel sensitivity: POOR METABOLIZER, REDUCED ANTI-PLATELET EFFECT - gene: CYP2C19 - gene result *2/*2.” Based on this recommendation, the physician may adjust the dose accordingly or choose another drug. In this case, the physician will likely increase the dose of clopidogrel in order to achieve therapeutic effect. Reprinted with permission from [65].

On an individual scale, the derivation of clinically actionable pharmacological information from the genome is already a reality: the clinical annotation of a patient's full genome sequence has suggested the patient's likely resistance to clopidogrel, positive response to lipid-lowering drugs, and lower initial dose requirement of warfarin [37]. Thus, physicians will use pharmacogenomics alongside traditional clinical practices to predict which drugs are more or less likely to work, which patients will require more or less medication to achieve therapeutic response, and which drugs should be avoided on basis of adverse events. In order to achieve these goals, the findings of the research lab needs to be translated into the clinic, and the practice of using pharmacogenomics must be integrated into the existing medical system (Figure 6).

### 5.1. Prescribing

When a physician treats a condition, there can be multiple approaches to that treatment. Currently, a physician considers clinical and social factors when choosing an approach, asking questions such as, “how is the patient's organ function?”, “have their been any past problems with this type of treatment?”, “how compliant will the patient be with one treatment versus another?”, and “for this kind of patient, what is the best evidence-based treatment?”. Based on his or her clinical experience, the physician then chooses a drug to use. If there are multiple treatments available, the physician will choose one and monitor the patient's progress. Having the ability to know which drugs will work best beforehand can improve care, because a physician will administer the best treatment and not waste time on a treatment that is likely to fail for a particular individual.

One area where gene-based prescribing is steadily advancing is in the area of cancer genomics. Cancer drugs generally have many toxic side effects, and in many cases of advanced cancers, physicians “guess and test” medications by prescribing them and monitoring progress. In addition, the very nature of cancer is “personal,” insofar as each specific cancer is caused by the unique sum of individual somatic mutations (that is, mutations that occur in the individual after birth and are not inherited or passed on). Certain “signatures” of cancers, or mutations that produce similar cancer phenotypes, allow for the grouping of cancers into distinctions, such as leukemia or lymphoma, but even these exhibit significant variability among classifications.

Thus, the ability to sequence and study the genomes of cancer cells of an individual can help identify the driving somatic mutations and provide a tool for rational drug choice. For example, the median survival for advanced or recurrent endometrial cancer is very poor, due to the fact that physicians treat empirically with chemotherapy, which may have no therapeutic benefits. Researchers studying mutations in the pathways of endometrial cancer cell lines found that response to doxorubicin, a chemotherapy used to treat endometrial cancer, was related to mutations in the Src pathways, which are involved in cell proliferation, motility, and survival. By pinpointing mutations in this pathway, the researchers were able to rationalize supplementing the drug regimen with the addition of SU6656, a drug that competitively inhibits the Src pathway, which increased the sensitivity of some of the cell lines to doxorubicin [38]. As cancers are typically characterized by a lack of error-correction mechanisms and inhibited apoptosis, such an approach is particularly important, as the initial failure of a chemotherapeutic drug allows time for a cancer to develop further mutations and spread further. In the future, interrogating cancer genomes could allow rational drug prescribing, decreasing the amount of time spent on ineffective therapies and increasing the number of successful cures.

Pharmacogenomics can also play a role in drug decisions for prevalent conditions, allowing physicians to predict when a commonly successful therapy may fail. For instance, there is an arsenal of drugs doctors can use to combat the co-morbidities of type II diabetes. These co-moribidities are usually cardiac risk factors, such as lipid abnormalities and high blood pressure: the cardiac risk factor conferred by type II diabetes is equivalent to that of a prior myocardial infarction in a nondiabetic individual. Presently, the physician chooses drugs based on his best clinical judgment and then monitors the outcome of the treatment. However, as the tolerance and efficacy of certain popularly prescribed drugs has been shown to be tied to genetics, such information could be used in prescription decisions. For instance, statins are a class of drugs that are inhibitors of HMG-CoA reductase, an enzyme that helps produce cholesterol in the liver. Thus, statins are given in an effort to lower cholesterol, particularly low-density lipoprotein (LDL) cholesterol, whose increased levels are a cardiac risk factor. Statins are often prescribed to patients with type II diabetes and high cholesterol in order to help them reach a more healthy cholesterol range. Even though studies have suggested genetic influences on statin efficacy and tolerance, such findings are not yet widely applied in clinical medicine.

One study found that in individuals with diabetes, variation in the HMG-coA reductase gene was associated with a decreased response to statin therapy. In this study, a significantly greater percentage of individuals heterozygous for the G minor allele of rs17238540 were unable to reach target cholesterol and triglyceride goals when compared to individuals homozygous for the major allele. Additionally, these individuals had a 13% smaller reduction in total cholesterol and a 27% smaller reduction in triglycerides. This is an example of just one variation in the HMG-coA gene; other variations certainly exist and can impact how well a patient responds to statins [39]. Another gene that has been found to affect response to statins is the APOE gene, which is associated with the regulation of total cholesterol and LDL cholesterol. There are several variants in this gene, and there are differences between how type II diabetic individuals carrying these variants respond to statins. For instance, the individuals homozygous for the E2 variant were all able to reach their target LDL cholesterol; however 32% of individuals homozygous for the E4 variant failed to reach target LDL cholesterol. Moreover, E2 variant homozygotes had a significantly greater lipid lower response to statins than some of the other variants. Thus, APOE is another gene that may be predictive of statin resistance or reduced efficacy. Knowledge of these genes could play a role in the future of drug prescribing, as physicians would be able to predict a priori if a drug was going to succeed or if another drug would be a better choice [40].

One major caveat of gene-based prescription decisions (as well as dosing, as discussed below) involves the applicability of a finding in one population to other populations (see above: Association Methods). While a pharmacogenomic effect may be true for a given population (with a certain genetic background, in animal model parlance), it may not directly apply to other populations due to unknown genetic factors, especially combinatorial effects. Because there is no current standard for translating a result between ethnicities, follow-up work is required for each specific pharmacogenomic interaction before it is applied in a clinical setting.

### 5.2. Adverse Drug Reactions

Another factor physicians need to consider when choosing a drug is the risk of adverse events, or any detrimental, unintended consequence of administering a drug at indicated clinical doses. In a milder form, an adverse event could be an allergic rash from penicillin. These events can also be much more intense: severe adverse drug reactions (SADRs) are those that can cause significant injury or even death, and are estimated to occur in about 2 million patients a year in the United States. In fact, SADRs are the fourth leading cause of death in the United States, with about 100,000 yearly deaths. Because of the impact of SADRs, scientists and physicians hope that the application of pharmacogenomics can help predict which patients are most susceptible to experiencing an SADR to a given drug. With this knowledge in hand, a physician can either more closely monitor these patients or choose an alternative therapy [41].

For instance, statins have been associated with a rare but incredibly severe adverse reaction: myopathy and rhabdomyolysis. A study looking at the possible genetic influences of this reaction found a SNP in the SLCO1B1 gene associated with this severe adverse drug reaction, with an odds ratio of 4.5 [42]. However, there are also cases of individuals who experience milder symptoms and develop statin intolerance. Some of these individuals experience an elevation in creatine kinase or alanine aminotransferase while on statins, indicating possible muscle or liver damage. A recent study found that the functional variants V174A and N130D in the SLCO1B1 gene, which encodes the organic anion transporting polypeptide OATP1B1, are predictive of statin intolerance [43]. OATP1B1 in these individuals has reduced maximal transport ability, possibly leading to higher levels of statins in the patient's blood. Currently, studies are underway to determine if there is a difference between the available statin drugs with regards to these pharmacogenetic components, in order to better inform physicians about the drug choices they make.

In the effort of applying pharmacogenetics in the clinic, trials have already shown that screening tests have clinical utility. For instance, abacavir, a nucleoside reverse-transcriptase inhibitor used to treat AIDS, causes a hypersensitivity reaction in 5 to 8% of patients. This reaction can include fever, rash, and gastrointestinal or respiratory symptoms. Since this adverse reaction necessitates stopping therapy (and patients cannot be put on the drug again because of the risk of a more severe reaction upon re-exposure), physicians could avoid prescribing this drug if they were capable of predicting which individuals would have a reaction. Recently, it was identified that HLA-B*5701 was associated with hypersensitivity to abacavir. Armed with this information, a double-blind, randomized, prospective study in nearly 2000 patients was conducted to determine if screening for this variant could help prevent hypersensitivity reactions in AIDS patients. The results supported the use of pharmacogenetics in the clinic: prescreening eliminated immunologically confirmed hypersensitivity reactions and significantly decreased hypersensitivity symptoms, compared to the control group [44].

### 5.3. Dosing

Once a physician has chosen a drug based on efficacy and consideration of adverse events, the next step is to determine what the correct dose at which to administer the drug. Currently, clinical factors such as gender, weight, and kidney or liver function may be taken into account when dosing a medication. However, genetics can play a large role in how a drug is dosed as well.

As mentioned previously, a major reason drug doses differ between individuals is due to polymorphisms in proteins involved in pharmacokinetics or pharmacodynamics. Variation in enzymes involved in pharmacokinetics, such as the Cytochrome P450 metabolic enzymes (and mainly, CYP2D6, CYP2C9, and CYP3A4), can affect the availability of drugs reaching their targets. Alternatively, the targets themselves (PD genes) can respond differently based on their specific structure.

One of the emerging examples of dosing based on genetics is the anticoagulant, warfarin. Prescriptions for warfarin number about 30 million cases annually and are indicated to prevent myocardial infarction, venous thrombosis, and cardioembolic stroke. However, the dose needed to achieve adequate anticoagulation can vary by as much as twentyfold between patients. Currently, physicians start with an initial dose and titrate (adjust) over time until the target international normalized ratio (INR), an indicator of anticoagulation, is reached. However, until the therapeutic dose is reached, there is the opportunity for over-coagulation, which leads to an increased risk of thromboembolic events, or under-dosing, which can lead to ineffectiveness, and thus, hemorrhaging and bleeding. The discovery of variants affecting warfarin dosing have led to the creation of algorithms that use clinical (such as weight and other drug status) and pharmacogenetic (variants in CYP2C9 and VKORC1; see above, PK and PD Interactions, respectively) information in order to predict a patient's optimal starting warfarin dose. One such dosing algorithm, produced by the International Warfarin Pharmacogenetic Consortium, was capable of predicting doses using a pharmacogenetic algorithm at a significantly more accurate rate than an algorithm using clinical factors alone [15]. However, one of the drawbacks is that these predictions are most accurate in a Caucasian population; additional research is needed in populations of different ancestries in order to produce a more broad-spanning pharmacogenetic algorithm.

### 5.4. Applying Pharmacogenomics in the Clinic

Though examples exist of how pharmacogenomics could impact prescribing drugs, predicting adverse events, and dosing drugs, the actual application of pharmacogenomics is just beginning to gain traction. As pharmacogenomics knowledge steadily increases and the infrastructure for its usage continually develops, the day when all physicians regularly apply genetics to drug dosing draws closer. The challenges that remain include surmounting regulatory hurdles, developing ways to continually update known findings, delivering knowledge to physicians, and integrating genomics into medicine. However, scientists have worked to address these challenges, and pharmacogenomics will likely serve as one of the first major clinical applications of personalized genomic medicine.

In the United States, the FDA regulates drugs and drug labels. Therefore, the communication between scientists and the FDA will be critical to the adoption of pharmacogenomic information on drug labels. Evaluation will depend on the trial design, sample size, reproducibility, and effect size [45]. One benefit of pharmacogenomics is that the associations between genetics and drug effects is more concrete and immediately applicable than in other translational bioinformatics concepts such as disease risk assessment, where scientists are struggling with “missing heritability” and combinations of moderate risks. Because of this, unlike other therapies, which require a randomized clinical trial in order to prove efficacy, the application of pharmacogenomic principles may not require the same level of scrutiny. Rather than providing some novel therapy, the vast majority of pharmacogenomic findings are simply supplementing physician knowledge about previously approved drugs. Physicians already utilize the clinical backgrounds of their patients (i.e. weight, gender, presumed organ function, drug interactions, compliance) when making decisions about drugs. As long as adding the variable of genetic information is non-inferior to the current standard of care, there should not be resistance to its implementation [46].

Once a biomarker is shown to be important, other decisions will have to be made: Should testing for the biomarker be required, or should it just be recommended? Socio-economic considerations along with the predictive value of the biomarker will need to be considered. At first pass, the use of pharmacogenomic data may be completely left to the clinician's judgment until the FDA has formalized its role in their application. Once a pharmacogenetic biomarker is approved, the drug's label will need to reflect the genetic components involved: biomarkers identifying the patient population that should receive the drug would be printed under “indication,” biomarkers related to drug mechanism may appear under the “clinical pharmacology” section, and biomarkers related to safety may be indicated in “adverse events.” The challenge for the FDA and clinicians alike will require vigilance about updating new information as the onslaught of pharmacogenetic associations continues to pour in [45].

Pharmacogenetic research continues to discover new drug-gene interactions. The volume of new findings exceeds the capabilities of any individual to parse. Thus, bioinformatics will have to play an integral role in the translation of the data to the bedside. Text mining (see Methods: Cheminformatics/NLP) will be instrumental to extracting structured data from the literature in order to update knowledge bases, such as PharmGKB. Ultimately, this knowledge will be integrated into a centralized database to make the information accessible to all.

In order to fully translate pharmacogenomics into the clinic, this information must be well integrated with the electronic medical system (Figure 6). Full adoption will require a curated, updated database with FDA or evidence-based approved drug-gene interactions that would be available for physicians to use in their medical practice. For example, PharmGKB is primarily used as a scientific tool for identifying drug-gene interactions. However, its clinical utility was shown when it was used to generate drug recommendations based on an individual's fully sequenced genome [37]. Such resources serve as the precursor to the systems that will be in place when all individuals have sequenced genomes readily available for physician use.

Finally, for pharmacogenomics to be widely applied, personal genomics needs to become ingrained into modern medicine. Physicians and patients must be educated as to the benefits of genomic medicine, in order to dispel any myths and to avoid ethical issues. Moreover, genetic testing facilities meeting the U.S. government's Clincial Laboratory Improvement Amendments (CLIA) certification requirements need to be established in order to provide patients with genomic data that is considered acceptable for clinical use. Finally, insurance companies must be on board to reimburse genetic testing. Since sequencing costs continue to drastically fall, the debates surrounding cost will soon become moot [46]. Thus, we are rapidly entering an age where every patient can have his or her genome available. With the availability of an individual's genome, a physician looking to administer a drug such as a statin can check to see whether or not the statin would be expected to work and if any possible adverse events might be expected (Figure 6).

Pharmacogenetics is a rapidly developing field; however, some challenges remain in implementing scientific findings from the bench to the bedside. Because of the continued development and work in this field, these challenges will be addressed, ushering in an age of personalized drug treatments.

## 6. Summary

Pharmacogenomics encompasses the interaction between human genetics and drugs, which can be affected by variation in genes involved in pharmacokinetics (PK) and pharmacodynamics (PD). Thus, a major goal of pharmacogenomics is to elucidate which genes affect drug action, using cheminformatics, expression studies, and genome-wide association studies (GWAS). Association methods can be used to discover novel associations by comparing the genetic differences between cases with a certain phenotype and controls. Expression analysis and cheminformatics can be used to expand knowledge about drug-gene interactions by comparing gene expression or interaction profiles among drugs and genes. Analysis of these studies can yield information about how these genes affect drug action. Because of differences in haplotype structure between populations, studies validated in one population may not be directly applicable to a different population. However, as knowledge accumulates about drug-gene interactions, scientists can contribute to databases, such as PharmGKB, documenting known relationships (Table 1). As the volume of knowledge grows, text mining methods may become instrumental in interrogating the literature and collecting relevant data for clinical use. The application of pharmacogenomics in the clinic can help inform physicians in drug prescribing, drug dosing, and prediction of adverse events. Because many of the drugs undergoing pharmacogenomic study are already FDA-approved, adoption of pharmacogenomics in the clinic is mostly dependent on the availability of genome sequencing and the development of implementation infrastructure. Moreover, pharmacogenomics can also aid in drug development, providing pharmaceutical companies with an additional tool to design more successful, cheaper trials. Thus, pharmacogenomics promises to help launch medicine and drug development into the realm of personalized care.

**Table 1:** Examples of pharmacogenomics used in this chapter. Additional examples can be found at PharmGKB.

Drug	Gene (Selected Examples)	SNPs/Genotypes (Selected Examples)	Sources
Mercaptopurine	Inosine triphosphate, pyrophosphatase (ITPA), Thiopurine methyltransferase (TMPT)	rs41320251, rs1800584	[63], [62]
Succinylcholine	Butyrylcholinesterase (BCHE)	rs28933390, rs28933389	[61]
Perhexiline	Cytochrome P450 2D6 (CYP2D6)	CYP2D6 *4/*5, *5/*6, *4/*6	[60]
Clopidogrel	Cytochrome P450 2C19 (CYP2C19)	rs4244285	[59]
Albuterol	Beta-2 adrenergic receptor (ADRB2)	rs1042713	[58]
Metoprolol	Beta-1 adrenergic receptor (ADRB1)	rs1801252	[57]
Methotrexate	Methylenetetrahydrofolate reductase (MTHFR)	rs4846051	[56]
Warfarin	Cytochrome P450 2C9 (CYP2C9), Vitamin K expodide reductase (VKORC1), Calumenin (CALU)	rs1799853, rs1057910, rs7294, rs9934438, rs9923231, rs339097	[55], [54], [53], [52], [25], [51]
Atorvastatin	P-glycoprotein (ABCB1)	rs1045642, rs2032582	[50]
Statins	HMG-coA reductase (HMGCR), Apolipoprotein E (APOE), Solute carrier organic anion transporter family, member 1B1 (SLCO1B1)	rs17238540, APOE - E2, E4, rs4149056, rs2306283	[39], [40], [49], [43]
Abacavir	HLA-B*5701 genes	rs2395029, rs3093726	[48]

## 7. Exercises

(A) Download a genotype and phenotype dataset of your choosing. Using PLINK (http://pngu.mgh.harvard.edu/~purcell/plink/) or a statistical program such as R (http://www.r-project.org/), calculate the association (using a Fisher's exact test) between <Trait> and each SNP. After Bonferroni correction, does any SNP reach genome-wide significance? (B) Does using a different correction method such as Benjamini or False Discovery Rate (FDR) result in any more significant SNPs?
(A) Use a pharmacogenomic database (such as PharmGKB) to find genes that may interact with metformin. (B) Are any of these genes known to interact with other drugs? Which drugs? (C) Bonus question: Are any of these drugs related (by structure or function) to metformin?
(A) Implement a warfarin dosing equation (e.g. the one found in [15]). If you have a personal genotype, input your information and calculate your optimal starting warfarin dose; otherwise, calculate the optimal dose (as predicted by both the clinical and pharmacogenetic algorithms) for a 66-year old Caucasian (175 cm, 75 kg), not taking amiodarone or enzyme inhibitors, who is rs9923231 TT and CYP2C9 *2/*2? (B) Would the clinical algorithm have over- or under-estimated his (or your) dose and what are the potential consequences of such an error?
You are a physician and would like to prescribe simvastatin. What parts of the genome would you want interrogated to know about prescribing this drug and why?
Read about the clinical uses of a whole genome or exome in healthy [37] and diseased [47] individuals. How can pharmacogenomics be directly applied in a clinical setting?

Answers to the Exercises can be found in Text S1.

**Further Reading**

- Altman RB, Flockhart D, Goldstein DB (2012) Principles of pharmacogenetics and pharmacogenomics. Cambridge: Cambridge University Press. 400 p.
- Altman RB, Kroemer HK, McCarty CA, Ratain MJ, Roden D (2010) Pharmacogenomics: will the promise be fulfilled? Nat Rev Genet 12: 69–73.
- Altman RB (2011) Pharmacogenomics: ‘noninferiority’ is sufficient for initial implementation. Clin Pharmacol Ther 89: 348–350.
- Klein TE, Chang JT, Cho MK, Easton KL, Fergerson R, et al. (2001) Integrating genotype and phenotype information: an overview of the PharmGKB project. Pharmacogenetics Research Network and Knowledge Base. Pharmacogenomics J 1: 167–170.
- Roses AD (2000) Pharmacogenetics and the practice of medicine. Nature 405: 857–865.
- Roses AD (2004) Pharmacogenetics and drug development: the path to safer and more effective drugs. Nat Rev Genet 5: 645–656.

**Glossary**

- Adverse event - A “side effect,” or unintended consequence of taking a drug.
- Cheminformatics - Methods that utilize chemical structures of metabolites and/or protein structure to discover potential drug-gene interactions.
- Drug Target - The specific protein whose interaction with a drug constitutes that drug's mechanism of action.
- (Gene) Expression - The relative amount of RNA from a gene in a cell at a given snapshot in time, often used as a proxy for activity of the gene in the condition in which the experiment was performed.
- Hit - A small molecule that disrupts the function of a potential drug target (for treatment of a disease).
- Lead - An optimized (often chemically modified) “hit” with high specificity for its target and reasonable pharmacogenomic properties.
- Linkage - The property that multiple SNPs are often inherited together. When a SNP is associated with a trait or disease, it is not necessarily the causal SNP, but may be “linked” to other variation that is the molecular and physiological cause of the association.
- (DNA) Microarray - An experimental method that probes hundreds of thousands or millions of regions of the genome to determine the genotype at each locus.
- “Off-target” effect - The effects of a drug propagated by interactions with proteins other than the drug target (“innocent bystanders”).
- “On-target” effect - The effects of a drug propagated by the intended interaction with the drug target.
- Pharmacodynamics - The mechanisms that relate to “what the drug does to the body,” including “on-target” and “off-target” effects, intended and unintended, beneficial or harmful.
- Pharmacogenomics - The study and application of genetic factors relating to the body's response to drugs.
- Pharmacokinetics - The range of mechanisms that relate to “what the body does to the drug,” including absorption, distribution, metabolism, and elimination of a drug.
- Polymorphism - A mutation in the genome that varies among individuals in a sizable fraction (often, minor allele frequency >0.01) of the population.
- Polypharmacology - The interaction of a drug with multiple targets.
- SADR - Severe Adverse Drug Reaction. An adverse event that results in significant injury or death.
- SNP - Single Nucleotide Polymorphism (see Polymorphism)

## Supporting Information

Text S1 Answers to Exercises.
(DOCX)
