# Supplementary material for: Chapter 7: Pharmacogenomics
Source: PLoS Comput Biol. 2012 Dec 27;8(12):e1002817. doi: 10.1371/journal.pcbi.1002817 (PMC3531317; doi:10.1371/journal.pcbi.1002817)
Supplement: Text S1 — Answers to Exercises. (DOCX) [file pcbi.1002817.s001.docx]

**Answers to Exercises**

These exercises are intended as guides for the types of questions one might encounter while performing pharmacogenomics research. The questions are open-ended and there are no absolute answers to these questions: they may be subjective and in such a rapidly developing field, the resources and websites, and thus, the answers, are likely to change after publication. We provide the following hints to orient readers.

**1.** (A) Download a genotype and phenotype dataset of your choosing. Using PLINK (http://pngu.mgh.harvard.edu/~purcell/plink/) or a statistical program such as R (http://www.r-project.org/), calculate the association (using a Fisher’s exact test) between <Trait> and each SNP. After Bonferroni correction, does any SNP reach genome-wide significance? (B) Does using a different correction method such as Benjamini or False Discovery Rate (FDR) result in any more significant SNPs?

**Answer:** *Human genotype-phenotype datasets are often tightly controlled due to privacy concerns. Some are available by application through the Wellcome Trust (*[*https://www.wtccc.org.uk/info/access_to_data_samples.shtml*](https://www.wtccc.org.uk/info/access_to_data_samples.shtml)*) and dbGaP (*[*http://www.ncbi.nlm.nih.gov/gap*](http://www.ncbi.nlm.nih.gov/gap)*). Some mouse genotype and phenotype can be compiled from the Jackson Laboratories (*[*http://phenome.jax.org/*](http://phenome.jax.org/)*)*

**2.** (A) Use a pharmacogenomic database (such as PharmGKB) to find genes that may interact with metformin. (B) Are any of these genes known to interact with other drugs? Which drugs? (C) Bonus question: Are any of these drugs related (by structure or function) to metformin?

**Answer:** *Example genes: C11orf65, SLC22A1, PPARG. Answers may vary.*

**3.** (A) Implement a warfarin dosing equation (e.g. the one found in [15]). If you have a personal genotype, input your information and calculate your optimal starting warfarin dose; otherwise, calculate the optimal dose (as predicted by both the clinical and pharmacogenetic algorithms) for a 66-year old Caucasian (175 cm, 75 kg), not taking amiodarone or enzyme inhibitors, who is rs9923231 TT and CYP2C9 *2/*2? (B) Would the clinical algorithm have over- or under-estimated his (or your) dose and what are the potential consequences of such an error?

**Answer:** *(A): Using the calculator from the IWPC 2009 paper, this individual would be predicted to have a 14.13 mg/week dose. (B): Using the same calculator, the prediction using the clinical factors alone would be 31.14 mg/week, which would overestimate the dose (with the potential consequence of hemorrhaging).*

**4.** You are a physician and would like to prescribe simvastatin. What parts of the genome would you want interrogated to know about prescribing this drug and why?

**Answer:** *Example genes: SLCO1B1, HMGCR, CYP3A5. Answers may vary.*

**5.** Read about the clinical uses of a whole genome or exome in healthy [37] and diseased [47] individuals. How can pharmacogenomics be directly applied in a clinical setting?

**Answer:** *Answers may vary.*
